# CD300f immunoreceptor contributes to peripheral nerve regeneration by the modulation of macrophage inflammatory phenotype

**DOI:** 10.1186/s12974-015-0364-y

**Published:** 2015-08-12

**Authors:** Hugo Peluffo, Patricia Solari-Saquieres, Maria Luciana Negro-Demontel, Isaac Francos-Quijorna, Xavier Navarro, Ruben López-Vales, Joan Sayós, Natalia Lago

**Affiliations:** Neuroinflammation and Gene Therapy Laboratory, Institut Pasteur Montevideo, Mataojo 2020, CP 11400 Montevideo, Uruguay; Department of Histology and Embryology, Faculty of Medicine, UDELAR, Montevideo, Uruguay; Institute of Neurosciences and Department of Cell Biology, Physiology and Immunology, Universitat Autònoma de Barcelona, and Centro de Investigación Biomédica en Red sobre Enfermedades Neurodegenerativas (CIBERNED), Bellaterra, Spain; Immunobiology Group, CIBBIM-Nanomedicine Program, Hospital Universitari Vall d’Hebron, Institut de Recerca (VHIR), Universitat Autonoma de Barcelona, Barcelona, Spain; Neurodegeneration Laboratory, Institut Pasteur Montevideo, Montevideo, Uruguay

**Keywords:** Regeneration, Immunoreceptors, CD300, Macrophage M1/M2 phenotype, Schwann cell, Wallerian degeneration, Phagocytosis

## Abstract

**Background:**

It has recently become evident that activating/inhibitory cell surface immune receptors play a critical role in regulating immune and inflammatory processes in the central nervous system (CNS). The immunoreceptor CD300f expressed on monocytes, neutrophils, and mast cells modulates inflammation, phagocytosis, and outcome in models of autoimmune demyelination, allergy, and systemic lupus erythematosus. On the other hand, a finely regulated inflammatory response is essential to induce regeneration after injury to peripheral nerves since hematogenous macrophages, together with resident macrophages and de-differentiated Schwann cells, phagocyte distal axonal and myelin debris in a well-orchestrated inflammatory response. The possible roles and expression of CD300f and its ligands have not been reported under these conditions.

**Methods:**

By using quantitative PCR (QPCR) and CD300f-IgG2a fusion protein, we show the expression of CD300f and its ligands in the normal and crush injured sciatic nerve. The putative role of CD300f in peripheral nerve regeneration was analyzed by blocking receptor-ligand interaction with the same CD300f-IgG2a soluble receptor fusion protein in sciatic nerves of Thy1-YFP-H mice injected at the time of injury. Macrophage M1/M2 polarization phenotype was also analyzed by CD206 and iNOS expression.

**Results:**

We found an upregulation of CD300f mRNA and protein expression after injury. Moreover, the ligands are present in restricted membrane patches of Schwann cells, which remain stable after the lesion. The lesioned sciatic nerves of Thy1-YFP-H mice injected with a single dose of CD300f-IgG2a show long lasting effects on nerve regeneration characterized by a lower number of YFP-positive fibres growing into the tibial nerve after 10 days post lesion (dpl) and a delayed functional recovery when compared to PBS- or IgG2a-administered control groups. Animals treated with CD300f-IgG2a show at 10 dpl higher numbers of macrophages and CD206-positive cells and lower levels of iNOS expression than both control groups. At later time points (28 dpl), increased numbers of macrophages and iNOS expression occur.

**Conclusions:**

Taken together, these results show that the pair CD300f ligand is implicated in Wallerian degeneration and nerve regeneration by modulating both the influx and phenotype of macrophages.

## Introduction

Although axons in the peripheral nervous system (PNS) have the capacity to regenerate and reach distal targets after a mechanical injury, functional recovery is usually not complete [1]. Successful axonal regeneration and functional reinnervation depends on different factors such as severity and site of nerve injury, age of the subject, and the distance that axons have to grow until they reach distal targets, among others [2, 3].

After a peripheral nerve injury, the distal portion of the nerve undergoes progressive degeneration in a process called Wallerian degeneration (WD) [4]. While WD in the PNS is fast, taking 14–21 days to clear axonal and myelin debris, it is dramatically slow in the central nervous system (CNS) [5]. This fact has suggested that slow or deficient myelin and debris clearance from the injury site could create an inhibitory environment for axonal regeneration. Accordingly, Wld^s^ mutant mouse with a delayed WD shows impairment of axonal regeneration [6, 7]. Thus, endogenous or therapeutic compounds increasing the speed of WD might enhance axonal regeneration and target reinnervation. WD begins with axonal degeneration, followed by myelin ovoid breakdown and myelin clearance by Schwann cells and resident and infiltrating macrophages [8, 9]. The recruitment of resident macrophages to the injury site starts within hours while the infiltration of macrophages from blood begins 2–3 days after injury and peaks between 7 and 14 days [10, 11]. Finally, myelin clearance is complete from 8 to 14 days after nerve injury [12]. Some authors have classified WD in a two-stage process: the first one, an inflammatory process when pro-inflammatory cytokines such as IL-1β and TNFα are produced mainly by resident macrophages and Schwann cells, and a second stage of WD which aims at resolution of inflammation with secretion of anti-inflammatory cytokines such as IL-10 by infiltrated macrophages and Schwann cells [13–16]. New insights into macrophage activation related to macrophage polarization and their pro-inflammatory or anti-inflammatory responses have been reported [17, 18], and recently macrophages have been classified as M1 or “classically” activated macrophages and M2 or “alternatively activated” macrophages, depending on the profile of cytokines required for their activation [19, 20]. Taken together, these data suggest that macrophages involved in WD might be polarized to the M1 phenotype on the first stage and to the M2 phenotype for the resolution of inflammation. Different markers have been suggested to be representative of the different phenotypes, such as CD206 (mannose receptor) or arginase I for M2 and iNOS or IL-1β for M1 phenotype [20, 21]. Despite this important breakthrough in activated macrophage classification, only few studies have been published describing the M1/M2 macrophage phenotype after a peripheral nerve injury [22, 23]. Overall, the differences seen between macrophage phenotype in the PNS and CNS could contribute to explain the differences between the effective WD process in the PNS in comparison with the CNS where WD is very slow and inhibitory factors for nerve regeneration remain in the damaged tissue. Thus, modulation of inflammation and macrophage polarization to a M1-M2 phenotype may represent a strategy to promote regeneration in the PNS and the CNS. However, additional studies are needed to firmly establish this hypothesis.

Activating/inhibitory immune receptors like CD200R, TREM2, and SIGLECs have been shown to mediate important functions in checkpoints for the modulation of neuroinflammation [24, 25]. The CD300 family of activating/inhibitory receptors is composed in humans by six members that are able to form complexes on the cell surface through the interaction among their extracellular immunoglobulin domain [26–31]. Their combination in a complex differentially modulates the signaling outcome, suggesting a mechanism of how CD300 complexes could regulate the activation of myeloid cells upon interaction with their natural ligands [32]. All the CD300 family members share an extracellular region comprising a single Ig-like domain and were thought to have a myeloid lineage-restricted pattern of expression. However, the expression of CD300f was recently observed in microglia, oligodendrocytes, and neurons in vitro [33]. The importance of this family of receptors is highlighted by the fact that one of its members, CD300a, is the second gene with strongest evidence for positive selection between human and chimpanzee [34]. Moreover, CD300a and CD300f are among the genes with the highest upregulation after a spinal cord traumatic injury [33]. The CD300 family contains two inhibitory receptors, CD300a and CD300f, both displaying a long cytoplasmic tail with a variety of different tyrosine-based motifs, that are able to recruit phosphatases like SHP1 and SHP2 and therefore deliver inhibitory signals. The most interesting difference between these molecules, besides their different pattern of expression, is the existence of two binding motifs for the p85 subunit of PI3Kinase in the cytoplasmic tail of CD300f. In fact, it has been shown that CD300f delivers in vitro both inhibitory and activating signals, thus revealing a remarkable functional duality of this receptor [28, 35–38]. However, in vivo CD300f has shown to be mainly an inhibitory receptor, as shown in CD300f knockout animals using the experimental autoimmune encephalomyelitis (EAE) model of multiple sclerosis [39] and very recently in several models of allergy [40] and systemic lupus erythematosus [36]. This latter study shows that mouse CD300f (CLM-1) recognized outer membrane phosphatidylserine and regulated the clearance of apoptotic cells, being macrophages derived from CD300f knockout mice deficient for phagocytosis of apoptotic cells. Other recent reports suggest the existence of other main ligands for mouse CD300f, as phosphatidylcholine or ceramide [40, 41], and for human CD300f (IREM1), as sphingomyelin [42]. Despite the importance of CD300f in the regulation of inflammation and clearance of cell debris and apoptotic cells, no data is available regarding the expression of CD300f or its ligands in the normal and lesioned nerve and its role in regeneration.

In the present work, we characterize peripheral nerve expression of CD300f after a crush injury and the presence of its ligands. Moreover, by using soluble receptor fusion protein CD300f-IgG2a, we show that the blockade of the interaction between the immunoreceptor and its ligands impairs axonal regeneration and modulates macrophage M1/M2 phenotype.

## Materials and methods

### Animal surgery and treatment

Both male and female adult (8–12 weeks old) Thy1-yellow fluorescent protein mice, line H (Thy1-YFP-H; Jackson Laboratories, Bar Harbor, USA) [43], were used in these studies. All experimental procedures were approved by the IPMon Animal Care Committee and conducted according to international FELASA guidelines, national law, and ethical guidelines (Uruguayan Animal Care Committee).

The surgical procedure was carried out following sterile precautions. Mice were anesthetized with ketamine-xylazine (90–10 mg/kg) and the right sciatic nerve at the mid thigh level was exposed. Treatments were performed by direct injections at 45 mm from the tip of the third digit in 2 μl of sterile PBS, using a fine glass micropipette connected to a Hamilton syringe. Immediately after the injection and at the same position, the nerve was crushed in two different directions 30 s each time with fine forceps. The crush site was labeled with lamp black powder. The wound was closed with 5–0 mononylon Ethilon sutures (Ethicon) and disinfected. The sciatic and tibial nerves and the plantar skin were harvested at 24 h, 3, 10, and 28 days post lesion (dpl).

All nerve injections were performed in 2 μl PBS and at 10 μg/ml concentration of the following products: rCD300f-IgG2a (rat extracellular domain of CD300f fused to mouse IgG2a protein) or purified mouse myeloma IgG2a (Invitrogen, Cat. N° 026200).

### Histological and immunohistochemical procedures

At 10 and 28 days post lesion (dpl), mice were deeply anesthetized with pentobarbitone and intracardially perfused with saline followed by 4 % paraformaldehyde in 0.1 M phosphate buffer solution. The sciatic nerve, including its main tibial branch, was dissected to the ankle level and harvested. A sciatic nerve segment 3 mm distal to the injury site was postfixed in 4 % paraformaldehyde for 3 h, transferred to 30 % sucrose, and frozen for further immunohistochemistry procedures. The tibial nerve was sampled in two, one segment of approximately 14 mm was washed with PBS 0.01 M and whole mounted on slides in Mowiol mounting medium, whereas another segment of 2 mm at the ankle level was dissected out, postfixed in 2 % glutaraldehyde in 0.1 M phosphate buffer, and processed for embedding in Epon resin for semithin section preparations.

The sciatic nerve was cut longitudinally in the cryostat (8 μm thickness) and stored at −20 °C until used. Non-specific antibody binding was blocked with PBS 0.01 M + 1 % Triton + 10 % fetal bovine serum for 1 h at room temperature. Sections were then incubated overnight at room temperature with the following primary antibodies: rabbit anti-Iba-1 (1:3000; Wako 019-19741), rat anti-mouse CD206 (1:500; Serotec MCA2235), rabbit anti-iNOS (1:500; Calbiochem 482728), and rat anti-mouse F4/80 (1:150; Serotec MCA497). Macrophages were also demonstrated using biotinylated *Licopersicon esculentum* (tomato) lectin (6 μg/ml; L9389; Sigma-Aldrich). After washes with PBS-Triton 1 %, sections were incubated for detection with appropriate secondary antibodies (Invitrogen) and DAPI. Controls were made to rule out nonspecific staining by incubation without the primary antibody.

For the recognition of mouse CD300f ligand, immunohistochemical stainings using a soluble fusion protein containing the extracellular domain of rCD300f fused to the Fc region of the IgG2a mouse heavy chain or control mouse IgG2a were performed (both at 10 μg/ml). The studies were done in teased fibres and cryostat sections.

For immunohistochemistry of teased fibres, sciatic nerves were freshly dissected out and immediately immersed in 4 % paraformaldehyde in 0.1 M phosphate buffer for 3 h. After washing with PBS, the perineural sheath was removed and nerve bundles were separated using a pair of fine needles. Teased fibres were blocked with PBS 0.01 M + 1 % Triton + 10 % fetal bovine serum for 1 h at room temperature and then incubated with the following primary antibodies: rabbit anti-MBP (1:100; Sigma-Aldrich M3821), rat anti-S100 (1:200; Sigma-Aldrich HPA006462), and rCD300f-IgG2a (10 μg/ml), overnight at room temperature. After washes with PBS-Triton 1 %, sections were incubated for detection with appropriate secondary antibodies (Invitrogen) and DAPI.

For quantification of skin innervation, plantar pads of the hindpaw were removed at 28 dpl and processed as described [44]. Briefly, after being postfixed in 4 % paraformaldehyde and cryopreserved, 70-μm cryostat sections were obtained. Non-specific antibody binding was blocked with PBS 0.01 M + 0.3 % Triton + 1 % normal goat serum for 1 h at room temperature. Sections were then incubated in primary rabbit antiserum against protein gene product 9.5 (PGP9.5, 1:1000; Ultraclone) for 48 h at 4 °C. After several washes, sections were incubated for detection with appropriate secondary antibodies for 24 h at 4 °C and mounted on gelatin-coated slides. Five sections from each sample were used to quantify the number and density of nerve fibres present in the epidermis of the paw pads.

Tissue sections were examined using an Olympus IX81 microscope and images of the longitudinal sections were acquired at 20× with an AxioCam MRm Zeiss camera attached to a computer for further counts and imaging processing by using ImageJ software. Confocal images of teased fibres were acquired using a Leica TCS SP5 II confocal microscope.

Semithin sections (1 μm) were obtained from the tibial nerve blocks. Images of whole tibial nerve cross section were acquired at 10× with an AxioCam MRm Zeiss camera attached to a computer, while sets of images chosen by systematic random sampling of squares representing at least 30 % of the nerve cross-sectional area were acquired at 100×. Measurements of the cross-sectional area of the whole nerve as well as counts of the number of myelinated fibres were carried out by using ImageJ software.

### Flow cytometry

Cell surface expression of the CD300f (CLM-1) was tested by indirect immunofluorescence following standard techniques using a monoclonal anti-CLM-1 from hamster and the corresponding isotypic control [37, 39]. Cells from uninjured and crushed sciatic nerve were analyzed by flow cytometry at 3, 10, and 28 dpl as described previously [45] with some modifications. Briefly, animals were perfused with PBS to eliminate blood. Crushed sciatic nerves were harvested, cut in little pieces, and passed through a cell strainer of 70 μm and the cell suspension centrifuged. Samples were incubated with anti-mouse CD16/CD32 (1:100; Biolegend, Cat N°101319) for 15 min at 4 °C to block the nonspecific binding of immunoglobulins to the Fc receptors. Cells were incubated with CD45-PerCP (Biolegend), CD11b-PE-Cy7 (Biolegend), F4/80-APC (eBioscience), and monoclonal hamster anti-CLM-1 antibody (5 ug/mL) which was a generous gift from Genentech (San Francisco, CA) or an isotypic control (armenian IgG hamster from Serotec, Cat N° MCA2356), in PBS for 30 min at 4 °C. After washing in PBS, cells were incubated with an anti-armenian hamster IgG-FITC secondary antibody (Biolegend, Cat N°405502) in PBS for 30 min at 4 °C (dilution 1:100). Samples were analyzed with BD FACSCanto II Flow Cytometer and FlowJo Software (BD Biosciences).

### Evaluation of axonal regeneration

Tibial nerves from crushed sciatic nerves at 10 dpl were whole mounted onto microscope slides and coverslipped in Mowiol mounting medium. The number of YFP-positive fibres was visualized using an Olympus IX81 microscope. Regenerating axons were counted at 1-mm increments along the length of the tibial nerve beginning at 8–9 mm from the crush injury site. All evaluations were conducted by a researcher blinded to the treatment groups as described [46].

### Functional evaluation

The walking track sciatic functional index (SFI) test was also carried out to assess recovery of locomotor function. The plantar surface of the mouse hindpaws was painted with black ink prior to crossing a runway. Footprints corresponding to the operated and intact paws were easily identified. The print length (PL) and the distance between the first and fifth toes (toe spread, TS) and between the second and fourth toes (intermediate toe spread, IT) were measured. The three parameters were combined in the SFI [47] to quantify changes in walking patterns. The SFI varies between 0 (for uninjured) and −100 (for maximal impaired gait). The walking track test was carried out prior to surgery to obtain baseline scores and then on days 4, 7, 10, 14, 17, and 28 dpl to assess the recovery of locomotor function. A researcher blinded to the treatment groups conducted all evaluations.

### Isolation of RNA and QPCR

Previous to nerve harvesting, animals were perfused with ice-cold PBS to eliminate blood. Due to very low RNA recovery from each nerve, the RNA was isolated and purified from pooled homogenized nerves (from 1 mm proximal to 6 mm distal to the crush, *n* = 6 per group as described in [48] in TRIzol (SIGMA, T9424), and the aqueous phase was further purified using the Nucleospin RNA II Kit with RNase Free DNase treatment (Macherey Nagel 740955.50). RNA samples were reverse transcribed using M-MLV reverse transcriptase (Invitrogen 28025–013) and random primers. Quantitative PCR (QPCR) was performed using the following TaqMan reagents from Invitrogen/Applied Biosystems: TaqMan Fast Advanced Master Mix (1205919), exon-spanning probes for CD300f/CLM1 (Mm00467508_m1), IL-1b (Mm01336189_m1), iNOS (Mm00440502_m1), MRC1 (Mm00485148_m1), and IL-10 (Mm00439614_m1). The relative expression ratio is calculated using the real-time PCR efficiencies and the crossing point deviation of an unknown sample versus a control according to Pfaffl [49]. Eucariotic 18S RNA endogenous control (FAM-MGB 4333760) was included in the model to standardize each reaction run with respect to RNA integrity and sample loading. QPCR was performed using the Corbett Rotorgene 6000 apparatus and software. Cycling conditions were 50 °C for 2 min, 95 °C for 10 min, followed by 45 cycles at 95 °C for 15 s and 60 °C for 1 min. [48].

### Production of rCD300f-IgG2a

Chinese hamster ovary (CHO-K1) cells were stably transfected with pSecTag/mIgG2a constructs [26] and positive cells were selected with 250 mg/mL of Zeocin (Invivogen, San Diego, CA, USA). The chimerical protein was purified from the supernatant using a protein A-sepharose column (GE Healthcare, Pittsburgh, PA, USA) as described before [26].

### Phagocytosis assay

Ten days after the crush injury and the different treatments, mice were anesthetized and approximately 8 mm of the lesioned nerve distal to the injury site was obtained, epineurium dissected and discarded, and incubated in PBS + collagenase (2 mg/ml) for 30 min at 37 °C. After homogenization, single-cell suspensions from each nerve in separate wells were plated for 2 h in DMEM supplemented with 10 % fetal bovine serum and penicillin 100 U/ml, streptomycin 100 μg/ml and in the presence of fluorescent beads (1:1000; Life Technologies, F-8762). After several washes, cells were fixed in 4 % PFA and the number of beads per cell were quantified under epifluorescence microscope observation by a treatment-blinded researcher.

### Data processing and statistical analysis

All data are shown as mean ± standard error of the mean (SEM). Statistical analysis of behavioral data (SFI) was determined using two-way repeated measures ANOVA followed by Bonferroni post hoc analysis. One-way analysis of variance (ANOVA) followed by Tukey’s post hoc analysis was used for experimental data with more than two experimental groups. A value of *p* ≤ 0.05 was considered to be statistically significant.

## Results

### CD300f expression in the sciatic nerve after injury

**Fig. 1:**
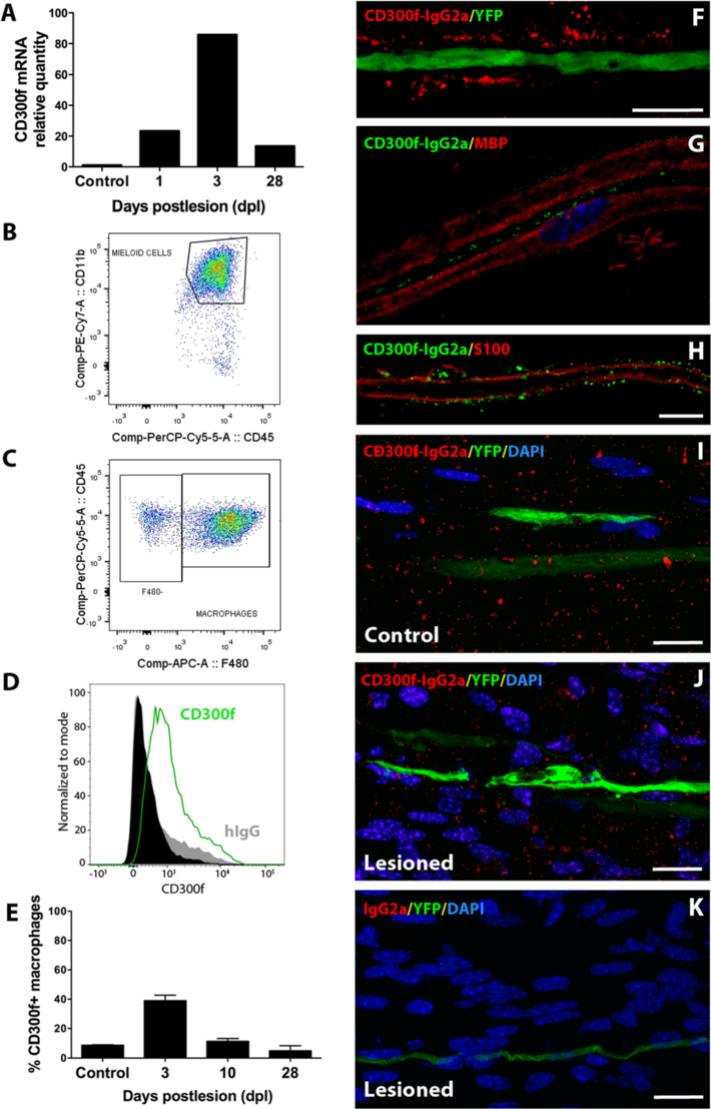
Expression of CD300f and its ligand in normal and lesioned nerve. QPCR from lesioned nerve show time-dependent increased CD300f expression (**a**, relative to unlesioned nerve = 1). **b-c**, representative flow cytometry profiles showing the selection of macrophages in the crushed sciatic nerve at 3 dpl (**b**, CD45^+^CD11b^+^; **c**, CD45^+^CD11b^+^F480^+^), and **d**, expression of CD300f in CD45^+^CD11b^+^F480^+^ cells in the crushed sciatic nerve at 3 dpl (*black*, only secondary antibody-FITC; *grey*, hamster isotype control; *green*, hamster anti-CLM-1). **e** Quantification showing the percentage of macrophages expressing CD300f in the uninjured nerve and at 3, 10, and 28 dpl. Note that CD300f peaked at 3 dpl declining thereafter. Confocal images of teased fibres from adult sciatic nerves showed staining for the CD300f ligand (using CD300f-IgG2a), which did not co-localize with the axon (**f**, Thy1-YFP-H mice) nor with the MBP-positive myelin domain of myelinating Schwann cells (**g**). Partial co-localization could be observed with the S100-positive non-myelinating outer limit of the myelinating Schwann cells (**h**). The staining with CD300f-IgG2a in uninjured nerve (**i**) did not change at 10 dpl (**j**). IgG2a negative control did no show any staining (**k**). Scale bar*s*: **b**: 30 μm; **c, d**: 100 μm; **e-g**: 20 μm

Expression of the immune receptor CD300f in the sciatic nerve was assessed by QPCR and flow cytometry in control uninjured and crush lesioned samples. Low basal CD300f mRNA expression was detected; its levels increased at 1 day post lesion (dpl), peaking at 3 dpl and declining thereafter, showing at 28 dpl slightly lower levels than at 1 dpl but still remaining high in comparison with uninjured nerves (Fig. 1a). Protein expression of CD300f and several macrophage markers (F4/80, CD45, and CD11b) were analyzed by flow cytometry in uninjured and crushed sciatic nerves at 3, 10, and 28 dpl (Fig. 1b–e). A small proportion of resident CD45+/CD11b+/F4/80+ macrophages expressed CD300f in the uninjured nerve (Fig. 1e). After crush sciatic nerve lesion, there was an increase in the total number of CD45+/CD11b+/F4/80+ macrophages present in the injured nerve in comparison with a non-injured nerve. Nearly 40 % of these macrophages expressed CD300f at 3 dpl, and the expression declined progressively to 28 dpl. Moreover, few F4/80 negative and CD11b+/CD45+ positive cells at 3 and 10 dpl also expressed CD300f, most probably representing neutrophils and maybe mast cells (not shown).

### CD300f ligand is expressed in the PNS

In order to characterize the presence of the ligand of CD300f in the PNS, we performed immunohistochemical studies using rCD300f-IgG2a fusion protein as described [50] on both frozen sections and teased fibres. Uninjured teased fibres from Thy1-YFP-H transgenic mice showed a punctate staining pattern with rCD300f-IgG2a, not co-localizing with the axonal cytoplasm (Fig. 1f). While no co-localization was observed at the MBP-positive myelin domain of Schwann cells (Fig. 1g), partial co-localization with the external limit of S100-positive Schwann cell domain was apparent (Fig. 1h). Frozen sections of crushed sciatic nerve were used in order to assess the expression of the ligand after the injury. At 10 dpl, no apparent difference was observed in the staining for the ligand in comparison with an uninjured nerve (Fig. 1i, j). No staining was detected using IgG2a negative control (Fig. 1k).

### CD300f participates in axonal regeneration

**Fig. 2:**
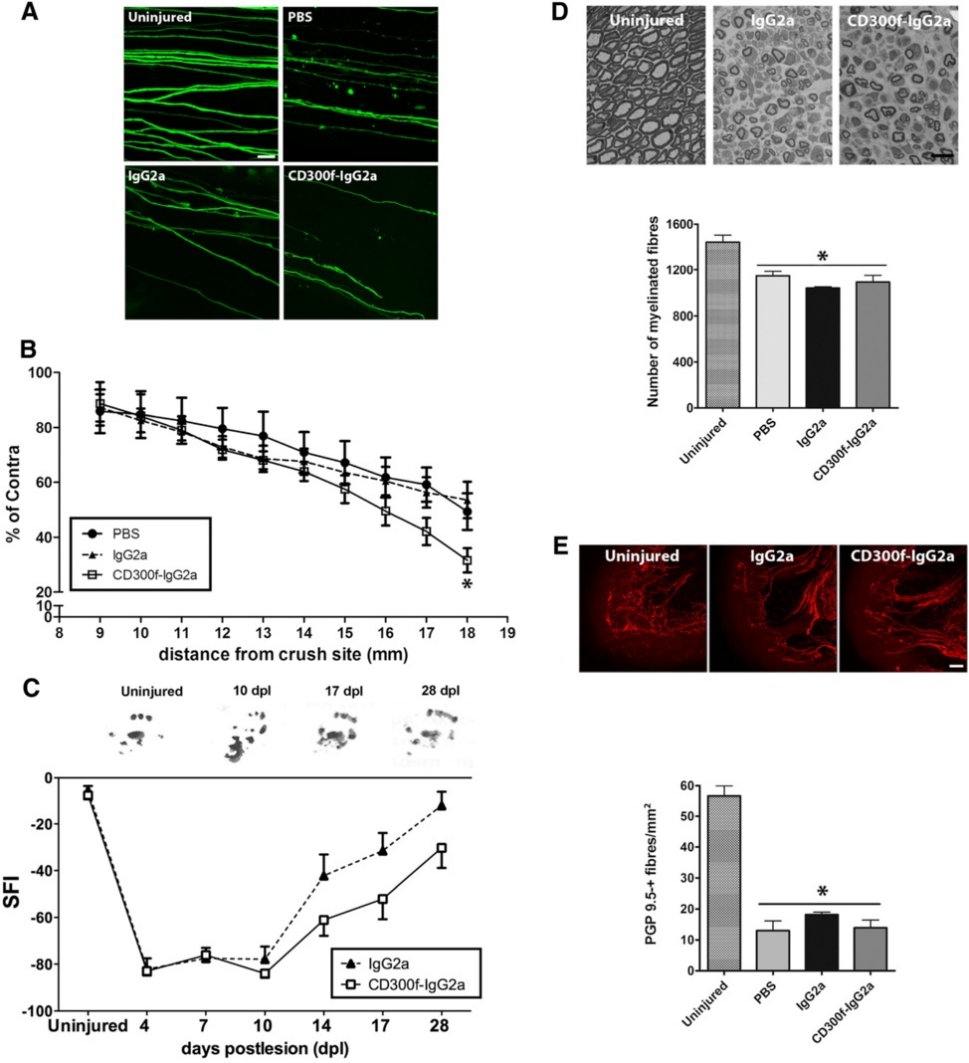
CD300f-IgG2a slows down axonal regeneration at 10 days after a sciatic nerve crush injury. Confocal images of whole mount tibial nerve at 18 mm distal from the crush site of uninjured or injured and injected nerves at 10 dpl (**a**). Thinner regenerating axons are seen in the tibial nerve (IgG2a and CD300f-IgG2a) compared with uninjured nerves. The group treated with the soluble receptor CD300f-IgG2a shows less axons growing distally through the tibial nerve than the IgG2a control group. **b** YFP-positive axons numbers are expressed as the percentage of contralateral axons at the indicated distance from the crush site at 10 dpl (*n* = 8 animals per group; *p < 0.05 vs. PBS and IgG2a). Representative footprints obtained from uninjured and at 10, 17, and 28 dpl are shown (**c**). The Sciatic Functional Index (SFI) walking track analysis revealed a strong tendency (*p* = 0.07) towards delayed functional recovery after treatment with CD300f-IgG2a when compared to IgG2a control animals (*n* = 8 mice per group). Representative micrographs of transverse sections of an uninjured sciatic nerve and at 28 days after crush injury and injection of IgG2a or CD300f-IgG2a show regenerated axons with thinner myelin at 28 dpl (**d**). Quantification shows a decreased number of myelinated axons in the tibial nerve with significantly fewer myelinated fibres in all crush injured animals (*p < 0.05 vs. uninjured group). The analysis of skin innervation at 28 dpl by the quantification of the number of intraepidermal nerve fibres in the plantar skin immunolabeled against protein gene product 9.5 (PGP 9.5) showed a significant reduced number of nerve fibres after crush injury, but no differences were observed between treated groups (*p < 0.05 vs. uninjured group). Scale bars: **a, e** 50 μm; **d** 10 μm

In order to evaluate the role of CD300f in regeneration after a crush nerve injury, the crushed sciatic nerve of Thy1-YFP-H transgenic mice was injected with a single dose of soluble CD300f-IgG2a at the moment of the injury. CD300f receptor-ligand interaction blocked in this way has been shown to render identical results compared to CD300f knockout animals [39, 40]. Axonal regeneration at 10 dpl was evaluated by counting the number of YFP-positive fibres growing through the tibial nerve as previously reported [46, 51]. In transgenic YFP-H mice, approximately 3 % of myelinated peripheral nerve fibres are YFP positive, with approximately 58 % of them being sensory axons and 42 % motor axons [52]. Consequently, Thy1-YFP-H mice have provided a valuable tool for studies of Wallerian degeneration [53] and nerve regeneration [46, 51, 52, 54]. When lesioned sciatic nerves were injected with CD300-IgG2a, a significant lower number of regenerating axons growing long distances were observed compared to both mIgG2a and PBS control groups (Fig. 2a, b). We further evaluated the recovery of function after crush injury by analyzing the hindpaw prints to obtain the SFI. After crush sciatic nerve injury, the SFI drops to −80 % whereas it recovers by day 28 up to −20 % with no significant differences when compared to pre-injury values. After CD300f-IgG2a treatment, a strong tendency (*p* = 0.07) towards delayed functional recovery was observed compared to IgG2a control animals (Fig. 2c). A detailed study of regenerated fibres was performed at 28 dpl by analyzing the number of regenerated myelinated fibres counted in semithin sections in the tibial nerve at the ankle level, and no differences between groups were observed (Fig. 2d). In addition, distal re-innervation was assessed by counting the number of PGP9.5-positive fibres in the epidermis. The number of PGP9.5-positive fibres was significantly lower in injured groups than in the contralateral skin at 28 dpl but no differences were seen between treatments (Fig. 2e). Taken together, these data show that a single injection of the soluble CD300f-IgG2a transiently delayed the regeneration, followed by endogenous compensations that achieve normal regeneration at longer time points.

### Blocking CD300f/ligand interaction induced macrophage accumulation and changes in phenotype

**Fig. 3:**
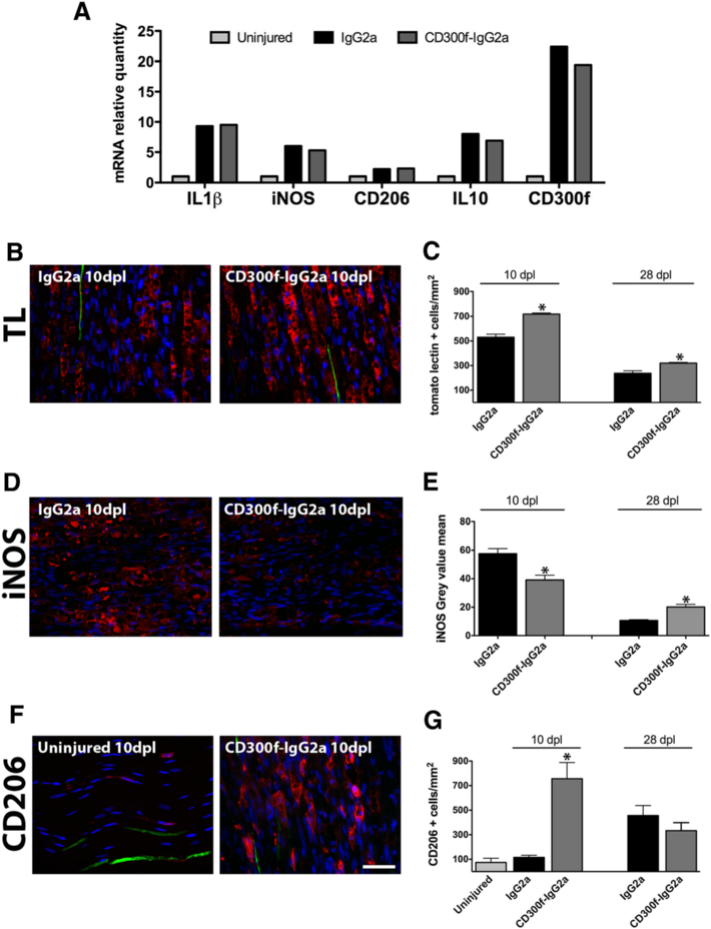
CD300f-IgG2a increases macrophage infiltration and induces their phenotypic change after a sciatic nerve crush injury. QPCR shows an increase of several cytokines and CD300f at 1 dpl with no differences between treatments (**a**). Infiltrated macrophages stained by tomato lectin show increased number at 10 and 28 dpl following CD300f-IgG2a treatment (**b, c**). The macrophage phenotype is altered after CD300f-IgG2a treatment, showing decreased iNOS staining at 10 dpl and increased staining at 28 dpl (**d, e**: mean grey value). On the contrary, the number of CD206-stained cells shows an important increase at 10 dpl (**f, g**), but no significant differences at 28 dpl. YFP-regenerated myelinated fibres can be seen in *green* in **b** and **f**. (*p < 0.05 vs. uninjured and IgG2a treatment). Scale bar in **b** and **e**: 40 μm

In order to understand the mechanism of impaired regeneration by blocking CD300f function, we assessed the level of inflammatory markers and macrophage infiltration and phenotype. QPCR from nerve samples at 1 dpl showed increased mRNA for IL-1β, iNOS, CD206, and IL-10 when compared to uninjured nerves (Fig. 3a). However, no significant differences were observed after the injection of CD300f-IgG2a or IgG2a. Moreover, the blockade of CD300f/ligand interaction did not alter CD300f mRNA at 1 dpl. Interestingly, later on at 10 dpl, immunohistochemistry against the general macrophage marker tomato lectin in the sciatic nerve distally to the crush showed increased numbers of positive cells with the typical morphology of phagocytic macrophages, which were further increased by treatment with CD300f-IgG2a (Fig. 3b, c). Similar results were obtained with anti-Iba-1 general macrophage marker (not shown). At 28 dpl, phagocytic macrophages could still be seen in the sciatic nerve although at lower numbers than at 10 dpl (Fig. 3c). However, in the group injected with CD300f-IgG2a, macrophages remained at significantly higher numbers than in the control group.

We further evaluated whether the blockade of the interaction between CD300f and its ligand had an effect in the phenotype of the macrophages that infiltrated into the nerve. We performed immunohistochemistry against the mannose receptor (CD206) as a marker of alternative M2 macrophages and against iNOS as a marker of classical proinflammatory M1 macrophages. iNOS labeling was significantly reduced in animals treated with rCD300f-IgG2a at 10 dpl but increased at 28 dpl in comparison with the injured control group (Fig. 3d, e).

CD206 labeling of the uninjured and IgG2a-treated nerves showed a low number of CD206-positive cells scattered through the nerve without differences between uninjured and injured nerves at 10 dpl. However, in the group of animals injected with rCD300-IgG2a, there was a significantly higher number of CD206-positive cells at 10 dpl (Fig. 3f, g). At 28 dpl, the injured control group showed higher numbers of CD206-positive cells in comparison with uninjured nerves and control injured nerves at 10 dpl but without significant differences between the injured control group and the rCD300f-IgG2a-treated group. Interestingly, considering all the experimental groups together, the number of regenerating YFP-positive fibres negatively correlated with the number of CD206-positive cells at 10 dpl (Spearman’s rank test, *p* = 0.02, *r* = −0.64). This correlation was not observed at 28 dpl.

**Fig. 4:**
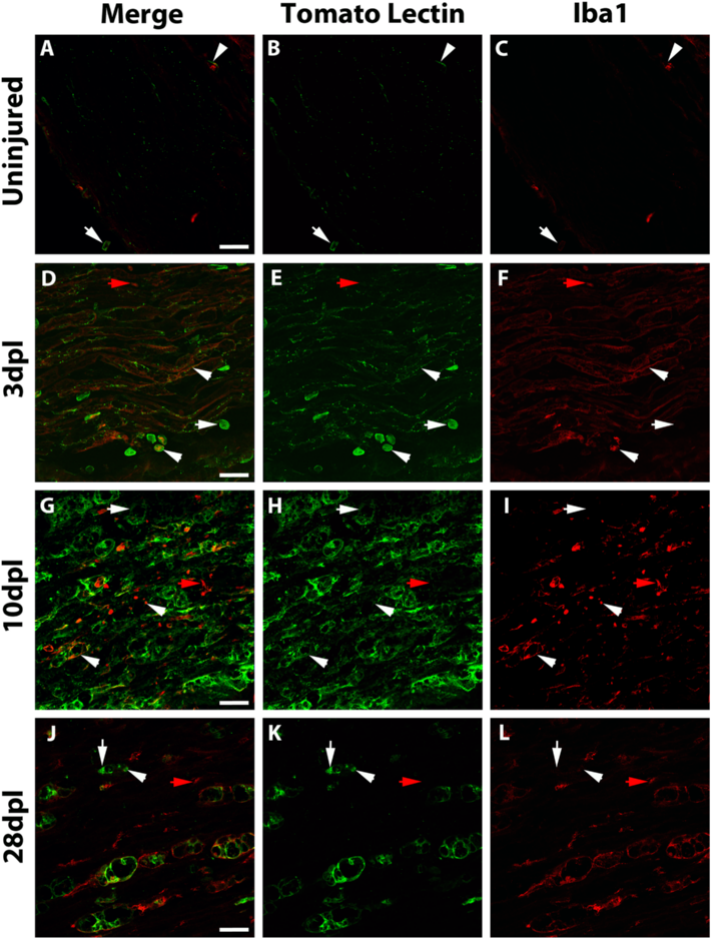
Tomato lectin stains most of the macrophages in the normal and injured nerve. Single focal plane confocal microscopy showed tomato lectin staining for macrophages in the normal and lesioned nerve. Tomato lectin and Iba-1 labeled the very few resident macrophages of the normal nerve (**a-c**, *arrowhead*) and meninges (**a-c** *arrow*). An increase of tomato lectin and Iba-1 staining was observed at earlier time points after a crush injury (3 dpl), which peaked at 10 dpl and decreased by 28 dpl (**d-l**). Most macrophages were stained with both markers (**a-l**, *arrowheads*), including big foamy macrophages and some smaller and round macrophages. Interestingly, some cells were only stained with tomato lectin (**d-l**, *white arrows*) and other only stained with Iba-1 (**d-l**, *red arrows*). Scale bar: 20 μm

**Fig. 5:**
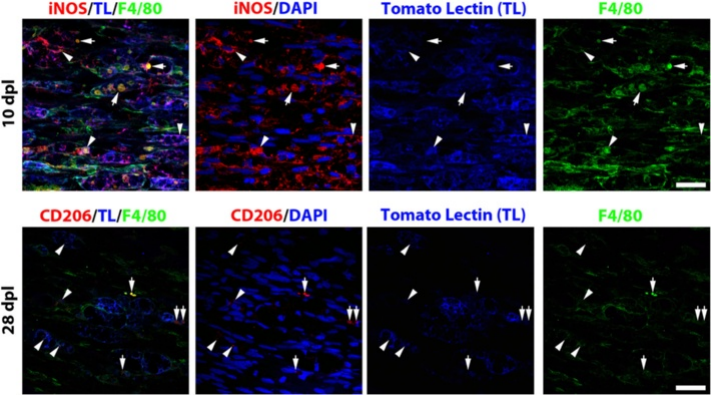
iNOS and CD206 stain a heterogeneous population of macrophages. Single focal plane confocal microscopy for iNOS or CD206 combined with tomato lectin and F4/80 macrophage staining evidenced that most cells co-stain with tomato lectin and F4/80, and some of them are in addition iNOS or CD206 positive (*arrowheads*). Moreover, there is a population of round and smaller F4/80-positive and tomato lectin negative macrophages that also shows iNOS or CD206 staining (*arrows*). Scale bar: 20 μm

Tomato lectin has been widely used as a marker of microglia/macrophages and endothelial cells in the CNS [55, 56]. Here, we show that tomato lectin is also a good marker for macrophages in the normal and lesioned nerve when compared to Iba-1 and F4/80 (Figs. 4 and 5). Tomato lectin labeling increased from 3 dpl peaking at 10–14 days post lesion (Fig. 4) as published by other authors using other macrophages markers such as F4/80 and CD11b [10, 11]. Tomato lectin and Iba-1 labeled the very few resident macrophages of the normal nerve (Fig. 4a–c). Interestingly, an important heterogeneity of macrophages was observed after the lesion. Most infiltrated large foamy macrophages at all time points were intensely stained with tomato lectin, Iba-1, and F4/80 markers (Figs. 4d–l and 5). However, smaller cells showed different combinations of stainings, including tomato lectin-positive and Iba-1 and F4/80-negative staining. This latter category would also include the endothelial cells. Moreover, both Iba-1 and F4/80 stained small macrophages that remained unstained with tomato lectin (Fig. 4d–i red arrows, Fig. 5 arrows). This macrophage phenotype heterogeneity could also be observed with M1/M2 markers like iNOS and CD206. Although most iNOS and CD206 staining co-localized with the big foamy tomato lectin and F4/80-positive macrophages (Fig. 5 arrowheads), round and smaller tomato lectin-negative and F4/80-positive macrophages also expressed iNOS or CD206 (Fig. 5 arrows). Taken together, these data show that all iNOS and CD206 staining was detected inside tomato lectin, Iba-1, or F4/80-positive cells.

### Phagocytosis after blockade of CD300f

**Fig. 6:**
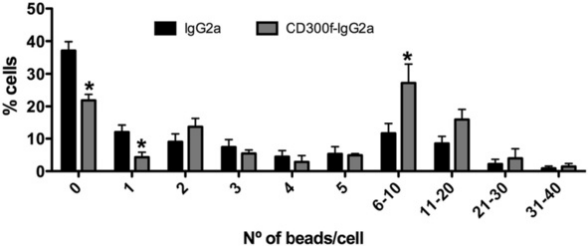
CD300f-IgG2a increases phagocytosis at 10 days after a sciatic nerve crush injury. Nerve cells acutely isolated from the injured nerve at 10 dpl show enhanced phagocytosis of fluorescent beads after CD300f-IgG2a treatment compared to control IgG2a. A significant decrease of cells having phagocyted none or 1 bead and an increase in the number of cells having phagocyted 6–10 beads was observed (*p < 0.05 vs. IgG2a treatment)

To further characterize the phenotype of inflammatory cells in the anti-regenerative milieu at 10 dpl induced by CD300f-IgG2a, we analyzed the phagocytic activity of nerve cells. Acutely isolated nerve cells were incubated for 2 h with fluorescent beads and the number of beads/cell was quantified. The treatment of the nerve with CD300f-IgG2a at the time of the lesion, induced 10 days later, increased phagocytic activity of nerve cells when compared to IgG2a treatment. A reduction in the percentage of cells with 0–1 phagocyted beads and an increase in the percentage of cells with 6–10 phagocyted beads were observed after CD300f-IgG2a treatment (Fig. 6).

## Discussion

In the present work, we show that both CD300f and its ligands are present in the non-injured peripheral nerve, and that CD300f mRNA and protein are increased after a crush lesion. Interestingly, a single injection of the CD300f-IgG2a soluble fusion protein into the injured sciatic nerve delays both axonal regeneration at 10 dpl and functional recovery but has no effects at long-term regeneration. This delayed regeneration is associated to a modulation in the number and phenotype of M1/M2 macrophages in the lesioned nerve.

Recent reports have shown that phospholipids as phosphatidylcholine or phosphatidylserine are ligands for CLM-1, the mouse orthologue of CD300f [35, 36, 41]. Phosphatidylserine/CLM-1 interaction contributes to apoptotic cell clearance and hence to dampen inflammation [35, 36]. Ceramide has also been described as a putative ligand for this receptor, contributing to dampening inflammatory reactions of mast cells in several allergy models by the activation of CLM-1 negative signaling [40]. The interaction of activating/inhibitory immunoreceptors with lipids appears to be a more general phenomenon [41]. For instance, some gangliosides and 3-O-sulfo-β-d-galactosylceramide (C24:1) are potential ligands for CD300b/CLM-7 [57]. In the central nervous system, staining of the brain and spinal cord with rat or human CD300f-IgG2a showed a distinctive punctuate pattern, mainly in oligodendrocytes of the white matter [50]. Accordingly, we show here a similar punctuate staining pattern with CD300f-IgG2a in peripheral nerves. Interestingly, by using teased nerve fibres and Thy1-YFP-H mice, we evidence the specific subcellular localization of the CD300f ligands to what appears to be the outer cell membrane of the non-myelinating S100-positive domain of myelinating Schwann cells previously described [58] and not to the MBP-positive myelin sheath or the axonal compartment. However, we cannot discard the possibility that the ligand may also be present in non-myelinating Schwann cells or some component of the extracellular matrix. Although electron microscope procedures are necessary to determine its precise location, our confocal images suggest the localization of the ligand in the outer non-myelin Schwann cell membrane [58]. The normal presence of the ligands in Schwann cells and oligodendrocytes point to supplementary roles in addition to the phosphatidylserine “eat me” signal or the ceramide-induced signaling previously described. Other authors have shown that CD200, the ligand for the inhibitory immune receptor CD200R, is expressed in Schwann cells in the intact nerve [59]. In the CNS, this receptor induces a tonic anti-inflammatory signal contributing to set the threshold and magnitude of proinflammatory signaling [24, 60]. Whether the ligand of CD300f expressed on Schwann cells and oligodendrocytes also contributes to the maintenance of this tonic anti-inflammatory state is an open question. Chang and co-workers showed that CD200 is downregulated after crush injury in the site of lesion [59]. They hypothesized that, after nerve injury, CD200 is downregulated in order to decrease immunosuppression and enhance influx of macrophages and the inflammatory response to eliminate myelin and axonal debris. The ligands for CD300f do not decrease at least at 10 dpl, suggesting different signaling mechanisms for these two receptors. Interestingly, despite intense invasion of macrophages into the nerve, a very similar staining pattern was observed for the CD300f ligands, suggesting that macrophages do not bear the ligand. In accordance, microglial cells in vitro did not stain with CD300f-IgG2a [50].

Recent findings suggest that the anti-inflammatory physiological state of most tissues is not only a passive state resulting from absence of inflammatory stimuli but an active condition that requires participation of several molecules responsible for the suppression of potentially inflammatory stimuli. Under this paradigm, a physiological function for the ligands of inhibitory receptors like CD200R, CD300a, or CD300f could be to contribute to the so-called “On” and “Off” signals [61]. “Off” signals are constitutively present in the brain parenchyma and are expressed mainly by healthy neurons, whereas “On” signals are expressed by endangered or impaired neurons [62]. The integration of all the inhibitory and activating inputs shapes the phenotype and response of microglial cells or macrophages accordingly. In fact, several activating and inhibitory receptors similar to CD300f like CD200R, TREM2, or SIGLECs have been reported to be key regulators of microglial and macrophage activation [24, 25]. Thus, these mechanisms, including CD300f and its ligands, could also be in place for the interaction of macrophages and Schwann cells in the normal and injured PNS to regulate the inflammatory status.

CD300f has been reported to deliver both activating and inhibiting signals [28, 35–37]. However, only inhibitory signals have been found on monocytic cell lines. For instance, crosslinking CD300f in the human THP1 monocytic cell line inhibited proinflammatory cell activation induced by several TLR ligands through a SHP1- and SHP2-mediated mechanism [63]. The role of CD300f in the proinflammatory activation of primary human monocytes/macrophages in vitro has not been reported. Using CD300f-deficient mice in the EAE mouse model, it was shown that CD300f acts as a negative regulator of myeloid cell activity by suppressing the production of inflammatory cytokines, nitric oxide, and demyelination [39], confirming the negative anti-inflammatory signaling exerted by CD300f on monocyte/macrophages. After nerve injury, we show that CD300f mRNA was increased from 1 dpl, peaking at 3 dpl, and decreasing at 28 dpl. The expression of the protein was demonstrated by flow cytometry. We found protein expression in a subpopulation of macrophages in non-injured nerves and also after a crush injury, where the expression peaked at 3 dpl. Interestingly, we observed few F4/80 negative cells that showed CD300f expression, a staining that could represent neutrophils or mast cells. Accordingly, CD300f expression has also been shown in neutrophils [26, 31] and mast cells [40], both of which participate in nerve injury and regeneration. The time window of appearance of CD300f positive cells correlates with the influx rate of monocyte/macrophages and neutrophils into the lesioned nerve [11, 64, 65]. To assess the possible role of CD300f and its ligands after a peripheral nerve injury, we took advantage of the description that CD300f receptor-ligand interaction could be blocked using CD300f-Fc fusion proteins, rendering identical results than those observed in CD300f knockout animals. For example, in the EAE model, CD300f-Fc worsened clinical scores to similar levels than CD300f KO mice [39, 40]. Moreover, intradermal pretreatment with CD300f-Fc enhanced passive cutaneous anaphylaxis responses in wild type but not in CD300f KO mice [40]. These results suggest that the effect of CD300f-Fc proteins is mediated by dampening CD300f signaling by an uncoupling of CD300f and its ligand rather than by directly activating signaling by the interaction of the soluble CD300f-Fc with the ligand. Interestingly, we show that dampening CD300f signaling using the soluble CD300f-IgG2a fusion protein-induced accumulation of macrophages that displayed an M2 alternative activation phenotype including increased CD206 and decreased iNOS expression. Whether this is a direct effect of dampening macrophage CD300f signaling or an indirect effect remains to be elucidated. Despite injection of the soluble receptor CD300f-IgG2a at the moment of the lesion, no acute changes on mRNA for CD206, iNOS, IL-1β, IL-10, or endogenous CD300f were observed at 1 dpl. This suggests a more complex and long lasting mechanism of Schwann cell and macrophage interactions determining the altered inflammatory response and delayed regeneration observed. In addition to the modulation of the proinflammatory phenotype, CD300f may also contribute to dampen inflammatory reactions promoting phagocytosis of apoptotic cells [36]. Phagocytosis of myelin and cell debris is a critical component of WD and successful regeneration [5]. The impaired regeneration after the single injection of CD300f-IgG2a might be related to inhibition of phagocytosis and thus delayed debris clearance. Accordingly, at 10 dpl, when no CD300f-IgG2a remains, the accumulation of debris may trigger the increased phagocytosis of nerve cells observed here that may be responsible for the delayed but successful regeneration at 28 dpl.

Despite the importance of macrophage phenotype in WD and axonal regeneration, only a few reports [22, 23] have described the expression of M1/M2 phenotypic cell markers after nerve injury and repair. In a recent paper, Ydens and colleagues made a description of the different markers of M1 and M2 macrophages after nerve transection and repair in mice, showing a rapid M2 polarization of macrophages after axotomy [22]. They evaluated a high number of markers of inflammation including iNOS, CD206, and IL-1β at different time points after nerve injury and mainly by QPCR. In accordance with our results, they observed a rapid fast induction of mRNA for IL-1β and IL-10 at 1 dpl. Moreover, they observed the upregulation of other M2 markers like Arg1, Ym1, or TREM2. They also reported that the mRNA for CD206 did not show changes at the different time points evaluated (until 14 dpl). In accordance, we did not observe notable changes in CD206 mRNA at 1 dpl or in protein level at 10 dpl in comparison with uninjured nerves. However, we also analyzed longer time points (28 dpl) to sample processes of resolution of the neuroinflammation and found an increase in CD206 staining in comparison with both uninjured and 10 dpl control injured sciatic nerves. This late increase might be a consequence of signals aimed to resolve inflammation by adjusting macrophage polarization towards a healing phenotype. In relation to M1 macrophage polarization after nerve injury, Ydens and colleagues did not show a significant change in iNOS, IL-12p40, or INFγ mRNA levels at the different time points post lesion studied. However, in the present work, we have seen a significant increase in the iNOS mRNA at 24 h after lesion and in the iNOS protein at 10 and 28 dpl. These differences in the results could be due to the type of nerve lesion used between the two studies, i.e., nerve section or nerve crush. Further experiments are needed to establish the effect of the different M1/M2 markers on nerve neuroinflammation and regeneration. In this line, we show that the manipulation of the CD300f/ligand interaction induces impairment of regeneration associated to important changes in M1/M2 markers. After a sciatic nerve crush injury, a single injection of CD300f-IgG2a significantly increased the number of tomato lectin-positive macrophages and CD206-positive cells and decreased iNOS immunoreactivity at 10 dpl, whereas an opposite effect was found at 28 dpl. These data suggest that blocking CD300f-ligand interactions not only contributes to an enhanced recruitment of macrophages but also to a change in the phenotype of normally recruited macrophages towards an early M2 phenotype, followed by a switch to a M1 phenotype of some macrophages later on. Interestingly, Mokarram and colleagues induced a nerve section followed by tubulization repair and IL-4 or INFγ treatment to polarize macrophages towards a M2 or M1 phenotype, respectively. Only IL-4 but not INFγ treatment induced increased Schwann cell migration, macrophage recruitment, macrophage polarization towards M2 phenotype, and regeneration [23]. In the absence of any treatment, they reported a main macrophage polarization towards an M1 phenotype at 21 dpl, while we show mainly a polarization towards an M2 phenotype at 28 dpl. Moreover, Mokarram and colleagues reported that the increase in CD206-positive cells at 21 dpl positively correlated with regeneration, while we observe in fact a negative correlation at 10 dpl and no correlation at 28 dpl. This apparent contradiction between both studies may be explained by the different nerve injury models, i.e., nerve crush versus section and tubulization, where the neuroinflammatory conditions are different and where the structural maintenance of the epi-, peri-, and endoneurium has strong effects. Moreover, the treatment with IL-4 may change fundamental endogenous neuroinflammatory mechanisms influencing the final outcome of regeneration observed.

## Conclusions

Taken together, these results establish a role for CD300f in peripheral nerve injury, involving this immune receptor and its ligands in the regulation of neuroinflammation, M1/M2 macrophage recruitment and polarization, and nerve regeneration. Moreover, the ligands of CD300f most probably located in Schwann cells may constitute critical players that participate in Schwann cell-mediated interaction with macrophages. Further experiments are needed to better understand the mechanisms of action of CD300f in peripheral nerve neuroinflammation and regeneration and the putative role of other CD300f-expressing cells as mast cells or neutrophils. Moreover, additional work with other markers of M1/M2 phenotype has to be performed to unravel the phenotype of macrophages and their function after a peripheral nerve injury and how the absence of CD300f signaling might influence the pattern of inflammation. Finally, after a crush nerve injury, macrophages that take part in the first stage of WD (1–10 dpl) would be polarized mainly towards a pro-inflammatory M1 phenotype whereas the resolution of inflammation at later stages (15–30 dpl) would be driven predominantly by M2 macrophages.
